# Virtual Traffic Light Implementation on a Roadside Unit over 802.11p Wireless Access in Vehicular Environments

**DOI:** 10.3390/s22207699

**Published:** 2022-10-11

**Authors:** Robert Wong, Jack White, Sumanjit Gill, Shahab Tayeb

**Affiliations:** Department of Electrical and Computer Engineering, California State University, Fresno, CA 93740, USA

**Keywords:** IEEE 802.11p standard, wireless communication, Intelligent Transportation Systems, smart transportation, vehicle routing, vehicular automation, autonomous vehicles

## Abstract

Blind intersections have high accident rates due to the poor visibility of oncoming traffic, high traffic speeds, and lack of infrastructure (e.g., stoplights). These intersections are more commonplace in rural areas, where traffic infrastructure is less developed. The Internet of Vehicles (IoV) aims to address such safety concerns through a network of connected and autonomous vehicles (CAVs) that intercommunicate. This paper proposes a Road-Side Unit-based Virtual Intersection Management (RSU-VIM) over 802.11p system consisting of a Field-Programmable Gate Array (FPGA) lightweight RSU that is solar power-based and tailored to rural areas. The RSU utilizes the proposed RSU-VIM algorithm adapted from existing virtual traffic light methodologies to communicate with vehicles over IEEE 802.11p and facilitate intersection traffic, minimizing visibility issues. The implementation of the proposed system has a simulated cloud delay of 0.0841 s and an overall system delay of 0.4067 s with 98.611% reliability.

## 1. Introduction

**Figure 1:**
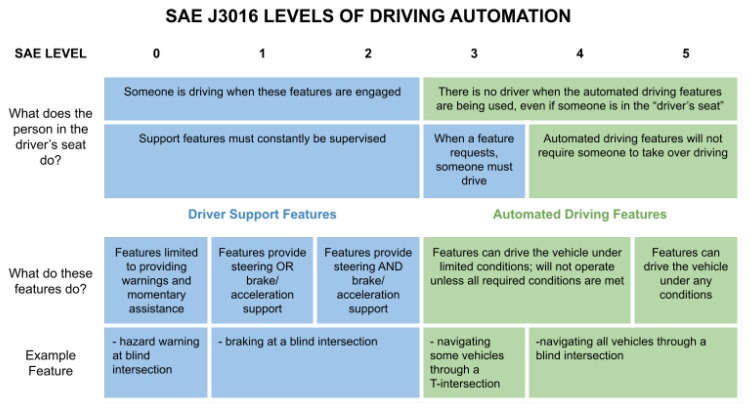
Levels of driving autonomy adapted from [1]. The example features are as related to the scenario of this paper.

Vehicular autonomy can be classified into six different levels: Level 0–Level 5 [1,2]. According to the Society of Automotive Engineers (SAE), Level 0 is ‘No Driving Automation.’ Level 1 is ‘Driver Assistance.’ Level 2 is ‘Partial Driving Automation.’ Level 3 is ‘Conditional Driving Automation.’ Level 4 is ‘High Driving Automation.’ Level 5 is ‘Full Driving Automation.’ A brief description of each level is available in Figure 1.

Various sensors and machine learning models may be developed and employed in vehicles to achieve the different levels of autonomy. Machine learning models are employed to control the vehicle and facilitate interactions with other CAVs and infrastructure [3]. The models will retrieve data collected by on-board sensors to make informed decisions [4]. Potential on-board sensors include: radio detection and ranging (radar), sound navigation and ranging (sonar), light detection and ranging (LiDAR), camera, and visible light communication [5]. Radar and sonar sensors use radio and sound waves, respectively, to detect objects and their distance from the sensor. Camera and LiDAR sensors require a direct line of sight to visually detect objects and their distance from the sensor.

Vehicular autonomy is enhanced with the introduction of inter-vehicular communications since it does not require line of sight for vision-based detectors [6]. Vehicles may communicate with each other and transmit relevant information regarding their velocity, acceleration, coordinate position, etc. This data may be used to expect and predict the positions of vehicles in the event that there is a lack of line of sight. Vehicles can directly communicate to one another through vehicle-to-vehicle (V2V) communication, or communicate facilitated by a roadside unit (RSU) via vehicle-to-roadside unit (V2R) communication such as in vehicle-to-infrastructure (V2I) communication [7]. V2V communication may be used in situations where there is little infrastructure and reduced traffic flow. Vehicles, at a greater resource cost (e.g., time and power consumed), will handle the communications and necessary computations themselves. V2R communication, however, can further assist in these scenarios by offloading the calculations and thereby reducing the amount of resources consumed by the vehicles [8]. On the other hand, reliable communication in a vehicular environment is difficult, even with standards such as 802.11p [9].

To further enhance traffic management by the added RSU(s), a virtual traffic light algorithm can be used. These algorithms replace physical traffic lights and directly communicate with vehicles to issue red and green lights in each traffic scenario [10]. When employed at the RSU-level in an optimized RSU, resource consumption and calculations may be even further offloaded from the vehicles. An area with a lack of infrastructure may indicate there is no connection to the power grid [11]. The RSU can operate and be powered off-grid through the use of solar energy.

### 1.1. Motivation and Research Gap

In blind intersections, vehicle on-board sensors will not have the necessary line of sight to detect vehicles that might be entering the interSection [12,13]. The proposed system addresses this issue by utilizing a low-cost, lightweight RSU to facilitate communication between the vehicles at the blind intersection. These characteristics are desired in order for operation in a rural area using an off-grid power system, e.g., via a solar panel, due to the lack of power grid connection in remote areas. The RSU will abide by the IEEE 802.11p protocol to communicate with vehicles so it may receive their positional, directional, and velocity-related data. The RSU will use this information to safely facilitate traffic through the intersection.

### 1.2. Contributions

The proposed system contributes the following to the body of research:

- Demonstrates the operability of RSU communication over 802.11p through the use of software defined radios.
- Adapts a virtual traffic light algorithm to offload computations from vehicles to an RSU using 802.11p.
- Presents and discusses the requirements for a remote, standalone, off-grid solar powered system to power lightweight RSUs, supported by power analysis and measurements.

## 2. Background

### 2.1. The Internet of Vehicles

**Figure 2:**
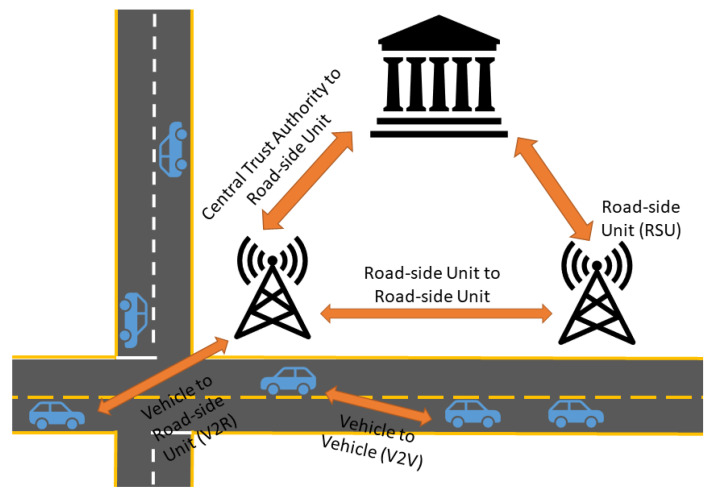
Hypothesized structure of the Internet of Vehicles adapted from [15].

The IoV is a network of CAVs that communicate with one another, as well as road-side infrastructure. It is hypothesized to function as Figure 2. As vehicles are driving, they will communicate relevant information (e.g., geographical location, speed, intention to change lanes, etc.) to one another. Road-side units are stationed throughout the network and maintain jurisdiction over a specific area. A central trust authority may also be present; this unit functions similarly to road-side units, only with a larger area to manage. These units communicate important information to vehicles, such as traffic accidents [14].

The central goal of the Internet of Vehicles is to transform society into a safer place for all participants. Reckless drivers will no longer be enabled to make decisions that endanger the lives of themselves and fellow citizens. This creates a safer form of transportation that will allow: pedestrians to safely cross the street, for all rules at intersections to be followed, for speed limits to be enforced, and increased safety in general. Optimal routing may also result from the IoV, enabling more efficient transportation in daily life. Emergency vehicles, such as ambulances and firetrucks, will be able to more quickly navigate to their destinations, as there will no longer be irresponsible drivers that ignore their sirens [16].

To implement the IoV, an important consideration that must be accounted for is the limitation on resource consumption especially time and power. If these resources are consumed quickly or misallocated, the network will become congested and may slow down, compromising its functionality [16]. As a result, optimizing communications is important for a more smooth flow of traffic. Another concern of implementation is security. The IoV is inherently vulnerable to cyber-attacks; these attacks may manifest as exploitation of the low security, in-vehicle controller area network (CAN) bus, or as man-in-the-middle attacks, replay attacks, etc. If these attacks were to remain unaddressed, severe consequences (e.g., injury, death) can occur for drivers and pedestrians. Therefore, security mechanisms must be put into place to preserve the goal of safety in the IoV [15]. In terms of securing the IoV, there are several exiting work that incorporate learning including [17,18].

### 2.2. Vehicular Communication

Vehicular communication requires standards to ensure that they meet safety requirements in a large network of fast moving vehicles, as well as operate on limited resources. Dedicated Short-Range Communication (DSRC) is wireless communication designed for automotive purposes, i.e., vehicle-to-vehicle (V2V) communication, vehicle-to-infrastructure (V2I) communication, and vehicle-to-everything (V2X) communication.

**Figure 3:**
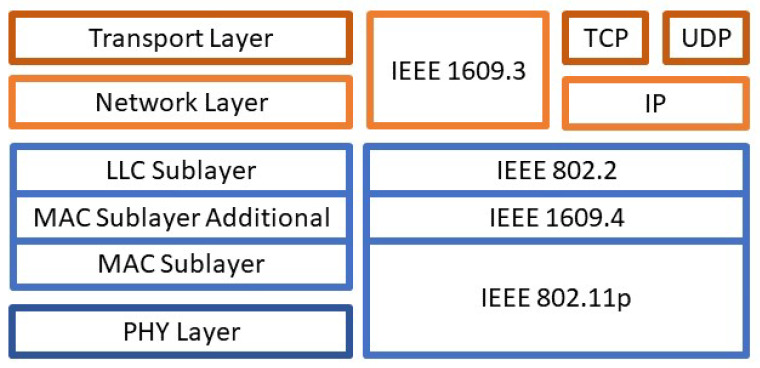
Visual representation of DSRC layers and protocols taken from [15].

DSRC is allocated the frequency band of 5.850–5.925 GHz for wireless communication in the Intelligent Transportation Systems (ITS) by the United States Federal Communications Commission (FCC) and the frequency band of 5.875–5.905 GHz by the European Union [19]. IEEE has also amended the 802.11 standard (Wi-Fi) as a part of DSRC for Wireless Access in Vehicular Environments (WAVE) for inter-vehicle wireless communication. The 802.11p standard was the original extension of 802.11 which defined the Physical Layer and the Data Link Layer of the communication and did not include exponential back-off or acknowledgements, but was later defined as IEEE standard 802.11-OCB (outside the context of a basic service set (BSS)), outside the context of a basic service set. Furthermore, WAVE utilizes IEEE 1609 which defines security services in IEEE 1609.2 and multi-channel operation in IEEE 1609.4 [19]. A depiction of the IEEE protocols in layers is shown in Figure 3 taken from [15]. The left side of the figure summarizes the main network layers separated by their color coding. The color coding on the right also summarizes what layer the protocol is on with some of them covering multiple layers.

Some of the mobility requirements vehicular networks need to support are speeds of around 200 km/h, a range of 1000 m, and communication within 100 ms [20]. V2I communication also allows for mobility management where vehicles report their position and trajectory to infrastructure that then predicts future positions [19]. Another protocol that supports the mobility of a vehicular network is Mobile Internet Protocol version 6 (IPv6). Mobile IPv6 defines a way for mobile nodes to maintain the same IP address even when moving between links, node operations, Internet Control Message Protocol (ICMP) messages, and more [21].

### 2.3. Virtual Traffic Lights

Virtual traffic lights propose to control traffic at locations such as intersections with no infrastructure using Vehicle-to-Vehicle communications [22]. In a vehicular network, vehicles are already communicating with each other, therefore a virtual traffic light will utilize the technology already used in vehicular communication. Algorithms have been implemented that effectively implement virtual traffic lights for certain intersections [23,24]. These papers also discuss the existence of locations where infrastructure for traffic lights is not feasible, and a virtual traffic light is a more cost-effective solution.

Garg et al. [25] proposed the use of a Deep Reinforcement Learning-based algorithm for virtual traffic light implementation. The model was a Deep Neural Network consisting of four layers coupled with a policy gradient algorithm. They verified its functionality on a custom simulator based on the Unity Virtual Reality platform and Python socket programming. Gohania et al. [26] applied an Artificial Immune System methodology to Virtual Traffic Light systems. This approach aimed to address how clusters of vehicles may be more readily sensed and handled by the leader vehicles elected by the Virtual Traffic Light algorithm. Wang et al. [27] introduced a 3-Level Buffer-based Virtual Traffic Light system that is coupled with an algorithm for collision avoidance so vehicles may more smoothly and reliably travel out of the intersection. They simulated their work and found that their approach minimizes average delay by 88% while decreasing congestion by 12%.

### 2.4. Roadside Units (RSUs)

Roadside Units (RSUs) are an integral part of communication for autonomous vehicles and facilitating network communications [14]. Research has been done on multiple aspects of RSUs, such as their placement and how they can support other mechanisms within the IoV [15].

The placement of RSUs has been investigated to optimize certain performance parameters, such as power consumption. Abdulrazzak et al. [28] proposed a method based on the Right-Side Left-Side model to distribute RSUs to minimize the amount of power consumed. Their approach was simulated in the SUMO simulation tool and led to a reduction in power consumption by 60% for low density traffic and 44% in high density traffic. Al Shareeda et al. [29] proposed the use of a genetic algorithm to determine the optimal number of RSUs as well as their placement in the vehicular network to maximize efficiency in dense traffic. They applied their approach to simulated traffic scenarios in Lebanon using the SUMO simulation tool and determined that three to four RSUs are ideally required in the proximity of dense traffic flow. Patra et al. [30] aimed to minimize both the power consumption and costs incurred by RSUs. They proposed a hybrid model that joins energy efficient RSU distribution algorithms and the scheduling of periods of sleep for RSUs and verified its functionality using the SUMO simulation tool.

Another researched feature is the provision of security mechanisms, such as authentication of vehicular communication [15]. Kovalev et al. [31] proposed a public key infrastructure scheme that employs the following security mechanisms: a handshake between the vehicle and the RSU, the signing of messages, and message authentication. They used the VEINS simulation tool to simulate traffic scenarios for the IoV and determined the introduction of their security scheme produces an overhead of 3.79 milliseconds [31]. Jolfaei et al. [32] proposed a novel security system for vehicle-to-RSU and RSU-to-RSU communications, wherein vehicles will be divided into groups and a single vehicle is elected to communicate with the RSU. This aimed to minimize the potential interception of outgoing communication of the RSU. The communications of spatial and temporal data between the lead vehicle and the RSU was further secured with the use of a lightweight, permutation-based encryption cipher to maintain its privacy and confidentiality [32]. Other works proposed reactive security schemes to address the compromising of RSUs. Abhishek et al. [33] aimed to mitigate data tampering as a result of malicious RSUs with a probabilistic model that uses authentication messages to determine the lack of synchronization between vehicles and the RSU. Their approach led to a reduction in latency by 7.5%, while maintaining a 99% detection rate of malicious nodes.

Another important aspect is offloading calculations to RSUs that can handle more computations compared to a mobile unit with limited resources. Since RSUs are stationary, they can be connected to grid systems, and can have more resource intensive components since they are not required to be mobile. Mobile units must reduce weight in order to more freely move, which reduces the number and size of components that are used. For instance, a smaller battery reduces weight in vehicles, but restricts the amount of power, and therefore restricts how much power can be used by certain tasks. Bazzi et al. proposed a virtual traffic light algorithm that is distributed among the vehicles at an intersection to determine which vehicle proceeds next [24]. Adapting the algorithm to be offloaded to some other device with less limited resources, such as an RSU, allows for more resources to be dedicated to other important features in an autonomous vehicle. Having some infrastructure increases costs negating one of the benefits of a distributed virtual traffic light, but a single RSU is more cost-effective than implementing a complete traffic light system.

## 3. Methodology

### 3.1. Traffic Scenario

**Figure 4:**
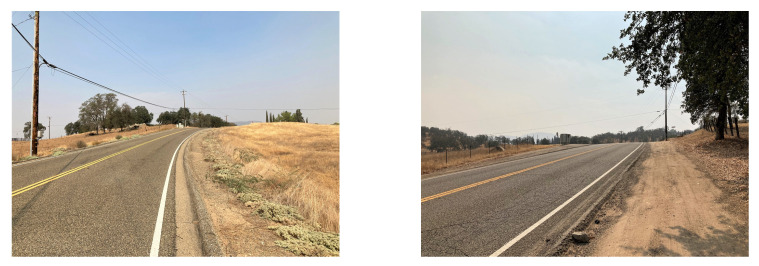
Example rural intersection from the point of view of vehicles traveling north–south through the intersection. Note the lack of visibility across the intersection.

**Figure 5:**
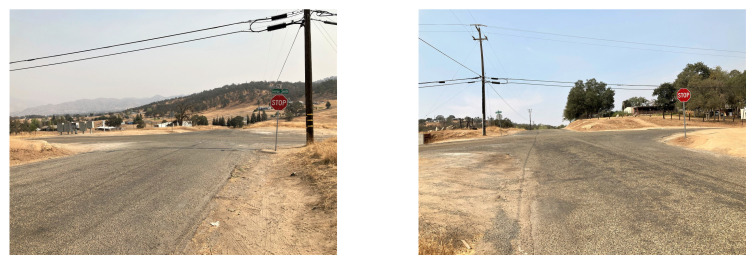
Example rural intersection from the point of view of vehicles traveling east–west through the intersection. Note that lack of visibility of traffic approaching in the north and south direction.

The proposed RSU aims to facilitate blind four-leg intersections in rural areas that are controlled by two way stop signs, but can be adapted for other intersection types. There is faster traffic on the main road and vehicles on the side roads must turn into oncoming traffic [12]. Figure 4 and Figure 5 illustrate sample blind intersections in a rural area. This is a blind intersection due to the side road’s positioning at the top of a hill that prevents line of sight from one side of the intersection to the other.

### 3.2. RSU Intersection Management

**Figure 6:**
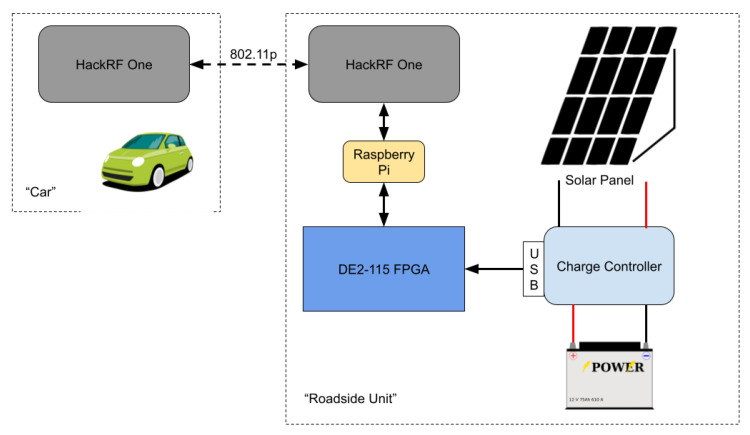
Block diagram of main components of the RSU and the test car.

The central component (refer to Figure 6) that will be stationed at the intersection is the road side unit (RSU). The RSU is modeled after virtual traffic light algorithms [24]. The RSU’s purpose is to take information about the location of vehicles and send information to the vehicles regarding whether or not it is safe to enter the intersection. Messages sent by the RSU will consist of simple payloads. The payload can be a single byte with parts that specify one of three options: a green light, a red light, or a request for intention of the direction a specific vehicle will be travelling. In response to a request, a vehicle will send two bits indicating which of the three directions (turning left, turning right, or going straight) the vehicle intends to follow.

Per the V2X Communications Message Set Dictionary defined by SAE J2735, vehicles should broadcast basic safety messages 10 times a second which includes a vehicle ID and position along with other information about the vehicle. In the proposed scenario, it is assumed that this includes the vehicle ID, and the x and y-coordinates of the vehicle. Once the RSU receives these broadcast messages, it will determine the lane leaders of each incoming lane where a lane leader is defined as a vehicle in the front of one of the four lanes entering the intersection. The RSU will then send an intention request to the lane leaders and receive a response from each of the vehicles using unicast messages. The RSU will then issue green or red lights based on priority and save intentions and light assignments as cached data until the vehicle has left the intersection. For the traffic on the side roads, it will alternate giving green lights between the two sides, and it is assumed that vehicles behind the lane leader will have mechanisms in place to keep a safe distance behind the lane leader.

**Figure 7:**
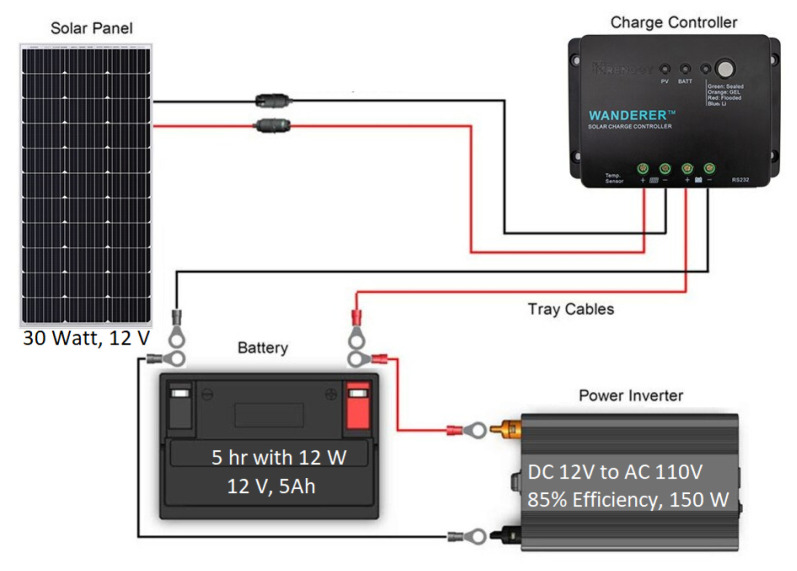
Main components for a small solar power system [34].

A remote power system is required for the RSU to operate without a connection to the power grid. Due to its lightweight nature, a solar panel system is selected. The system consists of a solar panel, charge controller, and a battery to conserve power for use when solar energy is not available. Figure 7 demonstrates how these components are connected. The inverter is not required if only DC voltages and currents are needed; however, the equipment for the RSU requires AC voltages for safe operation [34].

### 3.3. RSU-Based Virtual Intersection Management Algorithm

The RSU will send unicast messages to each of the leaders in the four lanes indicating whether they have received either a ‘green light’ (i.e., they are permitted to continue along their path) or a ‘red light’ (i.e., they must come to a stop and wait until the RSU issues a ‘green light’).

**Figure 8:**
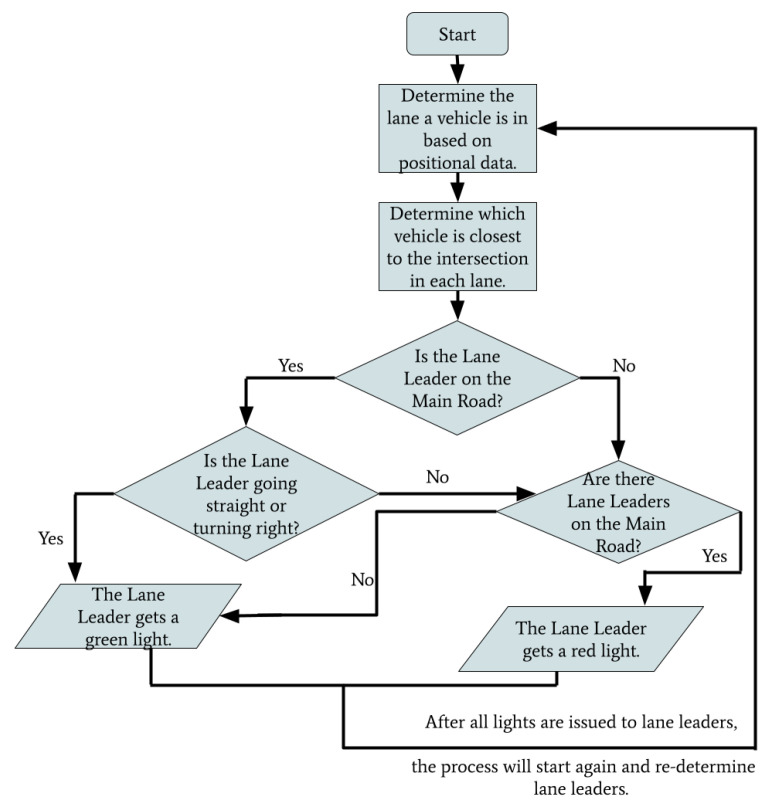
The above flowchart demonstrates the process used by the RSU to determine when to issue a vehicle a red light or a green light.

A flowchart depicting the general operation of the intersection management algorithm is shown in Figure 8. The leaders of each lane are determined in accordance with their location and proximity to the intersection. Based on the priority of the lane leader, they will be issued green or red lights. The process will then restart after all lights are issued and lane leaders will be re-determined. This loop will continue execution and address vehicles as they enter the intersection.

The technical operation of the algorithm is described in Algorithm 1 as pseudocode. The algorithm operates with an input of up to four vehicles who are the leaders of their respective lanes. The lane leading vehicles on the main road, i.e., in lanes one and two, are denoted as L1L and L2L. The lane leaders of the side roads, i.e., in lanes three and four, are denoted as L3L and L4L. The output of the algorithm is the collection of green and red light unicast messages that are issued to these leading vehicles.

When there are vehicles present on the main road, the algorithm operates as follows. If L1L is either going forward or turning right and L2L is also going forward or turning right, they are issued green lights, and the vehicles on the side roads are issued red lights. If one of the lane leaders on the main road is turning left and the other lane leader on the main road is either going forward or turning right, the vehicle turning left and the vehicles on the side road are given red lights. The other lane leader is granted a green light. If both vehicles on the main road intend to turn left, a tiebreaker determines which vehicle is given a green light, and which is given a red light. The vehicles on the side roads are issued red lights. If there is only one vehicle on the main road, it is issued a green light and the vehicles on the side roads are issued red lights.
**Algorithm 1** RSU Traffic Management.   **Input:** L1L, L2L, L3L, L4L   **Output:** Green Light and Red Light Unicast messages                                 for each Lane Leader**function** Manage-Traffic  **if** L1L **and** L2L go forward **or** turn right **then**      L1L and L2L get a green light      L3L and L4L get a red light  **else if** L1L **and** L2L turn left **then**      **if** L1L.ID≤L2L.ID **then**         L1L gets a green light         L2L gets a red light      **else**         L1L gets a red light         L2L gets a green light      **end if**      L3L and L4L get a red light  **else if** L1L turns left **and** L2L goes forward **or** turns right **then**      L1L gets a red light      L2L gets a green light      L3L and L4L get red lights  **else if** L2L turns left **and** L1L goes forward **or** turns right **then**      *L1L* gets a green light      *L2L* gets a red light      *L3L* and *L4L* get red lights  **else if** only L1L **or** L2L is in the intersection **then**      L1L **or** L2L gets a green light      L3L and L4L get a red light  **else if** only L3L is in the intersection **then**      L3L gets a green light  **else if** only L4L is in the intersection **then**      L4L gets a green light  **else if** L3L **and** L4L either go forward **or** turn right **then**      L3L and L4L get a green light  **else if** L3L goes forward **or** turns right **and** L4L turns left **then**      **if** L3L.ID≤L4L.ID **then**         L3L gets a green light         L4L gets a red light      **else**         L3L gets a red light         L4L gets a green light      **end if**  **else if** L4L goes forward **or** turns right **and** L3L turns left **then**      **if** L3L.ID≤L4L.ID **then**         L3L gets a green light         L4L gets a red light      **else**         L3L gets a red light         L4L gets a green light      **end if**  **else if** L3L **and** L4L turn left **then**      **if** L3L.ID≤L4L.ID **then**         L3L gets a green light         L4L gets a red light      **else**         L3L gets a red light         L4L gets a green light      **end if**  **else**      Do Nothing  **end if****end function**

If L3L and L4L are the only vehicles at the intersection, the algorithm operates similarly as if they were on the main road. If both vehicles on the side roads intend on going forward or turning right, they are issued green lights. If one of the vehicles on the side road intends to turn left and the other wants to continue forward or turn right, the vehicle that is turning left is given a red light. The other vehicle is granted a green light. If both vehicles are turning left, there is a tiebreaker that will determine which vehicle is given a green light and which is issued a red light. If there is only one vehicle on the side roads, it is given a green light.

### 3.4. Algorithmic Overhead

The overhead of the intersection management algorithm for the four lane leaders is determined to have a constant running time of Θ(1). There are up to four lane leaders in a given scenario. In the event that there are three to four lane leaders, the applicable scenario is found within the first five if-else-if statements. When there are no lane leaders, twelve if-statements will be examined before the final else-statement is reached and no further action occurs. The scenario that requires the largest amount of operations is when there are only two lane leaders, L3L and L4L, who are intending on turning left. There will be 13 if-statements that are examined and two additional statements to issue green and red lights to the vehicles. This will produce an overall running time of

(1) $$\begin{array}{cc} T(n)= & c_{0}+c_{1}+c_{2}+c_{3}+c_{4}+c_{5}+c_{6}+c_{7}\\ & +c_{8}+c_{9}+c_{1}0+c_{11}+c_{12}+c_{13}+c_{14}\\ & =\Theta (1). \end{array}$$

It should be noted that the expected input to the algorithm that manages the traffic is the four lane leaders. Therefore, the determination of lane leaders is not included in the algorithm’s running time. The running time of the process to determine lane leaders is computed as follows. When the vehicles broadcast their information, it must be added to a queue containing vehicular positional data. This queue will then be traversed to determine which lane each vehicle is in and if it is the closest to the intersection (i.e., it is the lane leader). This will require examining and operating on each of the elements in the queue, which will produce a linear running time of Θ(n), where *n* is the number of vehicles at the intersection.

The combination of the two processes of determining lane leaders and managing the intersection traffic produces an overall running time of: T(n)=Θ(n)+Θ(1)=Θ(n).

## 4. Experimentation

### 4.1. System Overview

**Figure 9:**
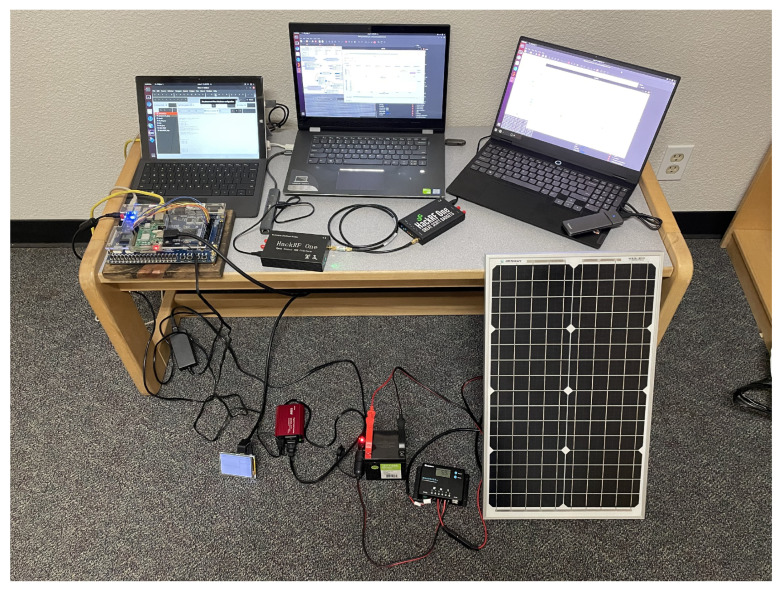
Layout of complete demonstration project. The leftmost laptop controls the DE2-115 FPGA board; the middle laptop controls the transmitting HackRF One SDR; the rightmost laptop controls the receiving HackRF One SDR.

The complete system consists of two HackRF One Software Defined Radios (SDRs) connected for the communication module, the DE2-115 FPGA for the RSU computational unit, the Raspberry Pi for cloud communication, and the solar panel system for a standalone power source. Figure 9 shows the complete setup with all of the components. Each individual part of the complete system is discussed in the following subsections.

#### 4.1.1. Communication Module

**Figure 10:**
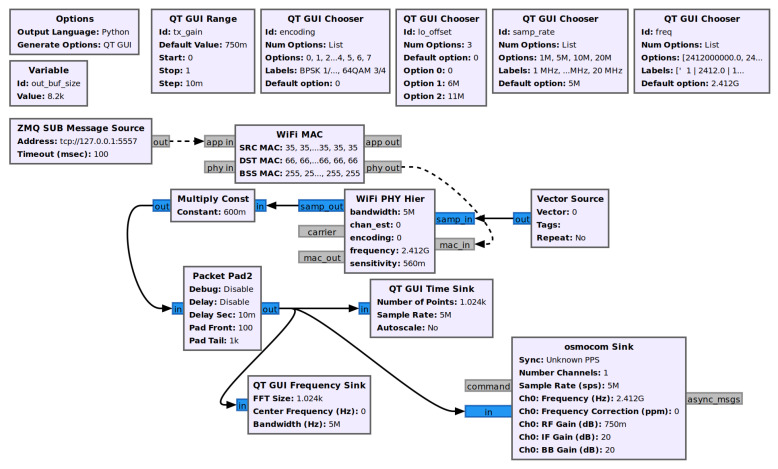
GNU Radio transmitter program flowchart for 802.11p. The messages are received through a ZMQ PUB-SUB communication, and the resulting 802.11p message is sent over the HackRF One using the osmocom sink.

**Figure 11:**
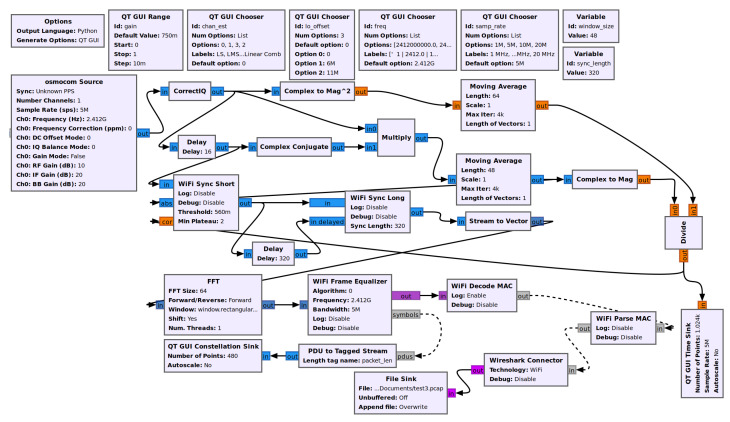
GNU Radio receiver program flowchart for 802.11p. The 802.11p messages are received through the HackRF One using the oscmocom source and decoded; resulting information and messages are saved to a pcap file for reading in Wireshark.

The communication module utilizes two Software Defined Radios, the previously mentioned HackRF Ones, that are connected by a coaxial cable. The cable is used for demonstration purposes. Transmitting a signal is practically the same, except does not broadcast over the open radio waves and does not potentially interfere with restricted frequencies. The communication module uses a GNU radio program to implement 802.11p on the SDRs. The communication is separated into two parts: the transmitter, and the receiver. The GNU Radio flowchart for the transmitter is shown in Figure 10. The GNU Radio flowchart for the receiver is shown in Figure 11.

**Figure 12:**
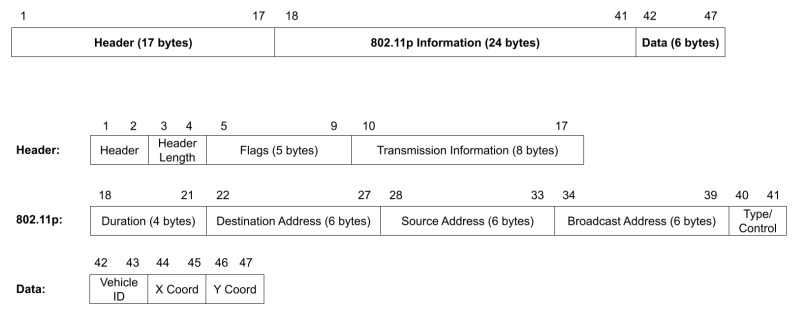
Layout of the transmitted 802.11p frame. The top demonstrates the general layout of the three main sections of the frame. Below that is each of the three sections expanded upon: the header, the 802.11p information, and finally the data.

The 802.11p frame constructed follows the format with the header fields and the source and destination addresses. The data sent consists of 3 components: the destination vehicle ID, the x coordinate, and the y coordinate, or flags that inform the vehicle to stop or proceed. A sample message is shown in Figure 12.

**Figure 13:**
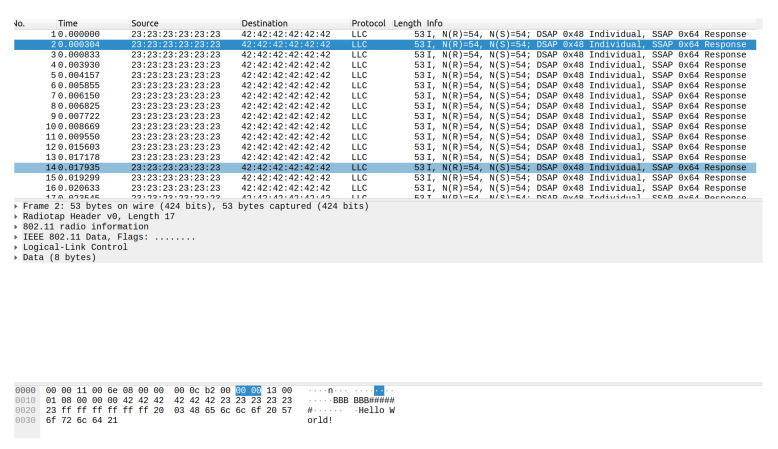
Example communication via 802.11p on Wireshark with test string “Hello World!”. The same string was continuously transmitted using the Message Strobe block.

The program makes use of several add-ons for GNU Radio. The WiFi blocks and general flowchart for the transceiver and receiver are adapted from [35]. This allows for sending and receiving messages in the 802.11 protocol format with the allowance for adjustments such as message size, frequency used, gain, and other variables. Additionally, to account for a DC offset in the transmitted signal on the receiving end, another add-on module is used, CorrectIQ [36]. Finally, the receiver program outputs the results to a .pcap file for readability through wireshark, an example file is shown in Figure 13.

#### 4.1.2. Cloud Server

In order to allow communication between the FPGA board and the GNU Radio program, an intermediary device is needed. The FPGA outputs data byte-by-byte to a Raspberry Pi board through the GPIO (general purpose input/output) pins, with an additional two bits that flag transitions between new bytes and new packets. The Raspberry Pi then simulates a cloud environment by sending the data across two ZMQ servers, the first connection being a PUSH-PULL connection and the second connection being a PUBLISHER-SUBSCRIBER connection. Via this connection, the DE2-115 FPGA board is able to send the information to the GNU Radio program via an intermediary device.

#### 4.1.3. Power System

**Figure 14:**
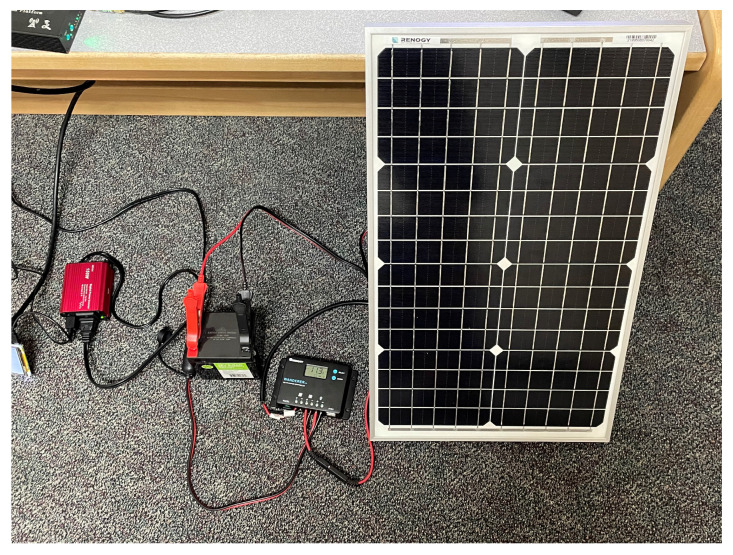
Standalone solar panel power system. The far left red block is the power inverter, to the right of that is the battery, then next is the charge controller, and finally on the far right is the solar panel.

Because access to power may be limited in rural areas, a solar powered system (refer to Figure 14) is used to provide power to the RSU. The power system requires an appropriately sized solar panel, battery, and charge controller to produce the necessary power to support the load. The load on this system includes the FPGA computational unit and the transmitting radio. The typical power requirements of each device in the system was measured to allow the calculations of solar panel and battery sizing for the level of autonomy required for critical systems. Since power equipment is expensive, a small system was developed for the design prototype that had a smaller panel and battery size than is required for full autonomy over multiple days.

## 5. Results

### 5.1. Traffic Management

**Figure 15:**
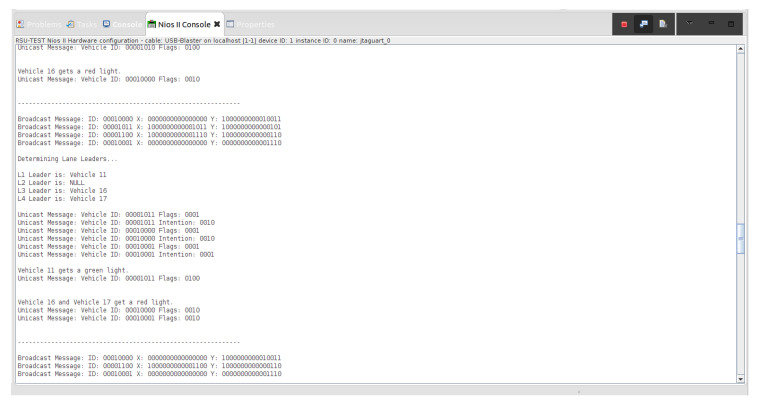
The above screenshot from the Nios II Eclipse Console demonstrates the successful traffic management of four vehicles at the blind intersection.

**Figure 16:**
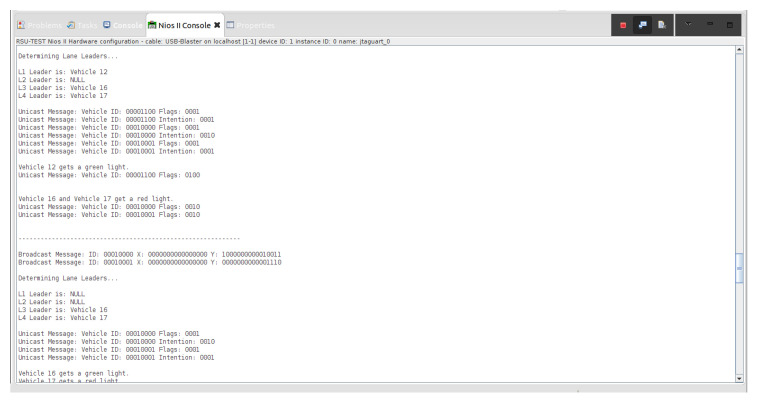
The above screenshot from the Nios II Eclipse Console demonstrates the successful traffic management for scenarios of three vehicles and two vehicles at the blind intersection.

**Figure 17:**
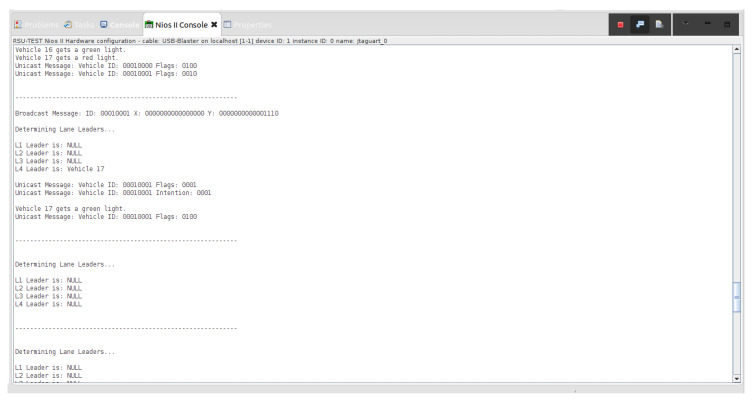
The above screenshot from the Nios II Eclipse Console demonstrates the successful traffic management of a single vehicle at the blind intersection.

Various traffic scenarios were simulated and successfully facilitated by the RSU. Four such scenarios are presented in Figure 15, Figure 16 and Figure 17.

Indicated in Figure 15, there are four vehicles at the intersection who are broadcasting their positional information to the RSU: Vehicle 16, Vehicle 11, Vehicle 12, and Vehicle 17. Vehicles 11, 16, and 17 are determined to be the lane leaders for Lane 1, Lane 3, and Lane 4, respectively. The RSU then issues unicast messages to the lane leaders asking for their intention. Vehicle 11 responds with a unicast message indicating it intends to turn left, Vehicle 16 states its intention to turn left, and Vehicle 17 states it plans to turn right. Because Vehicle 11 is on the main road and there is no vehicle in Lane 2, it is issued a green light. Vehicles 16 and 17 are issued red lights to wait until the intersection is clear.

There are then three vehicles in the intersection who transmit broadcast messages to the RSU: Vehicle 12, Vehicle 16, and Vehicle 17 (refer to Figure 16). These three vehicles are determined to be the lane leaders for Lane 1, Lane 3, and Lane 4, respectively. The RSU issues unicast messages to these vehicles to determine their intentions. Vehicle 12 responds by stating it intends to turn right, Vehicle 16 responds that its intention is to turn left, and Vehicle 17 responds that it intends on turning right. Vehicle 12 is the only vehicle on the main road and is issued a green light by the RSU. Vehicles 16 and 17 must continue to wait until the intersection is clear.

Vehicles 16 and 17 are then the only remaining cars in the intersection. They are identified as the lane leaders for Lane 3 and Lane 4, respectively. The RSU petitions the vehicles for their intentions, and they respond with unicast messages; Vehicle 16 states it wants to turn left and Vehicle 17 states it intends on turning right. A tiebreaker is used by the RSU to determine which of the vehicles will go onto the main road, since it is empty. Vehicle 16 is selected and is issued a green light, and Vehicle 17 is issued a red light.

**Figure 18:**
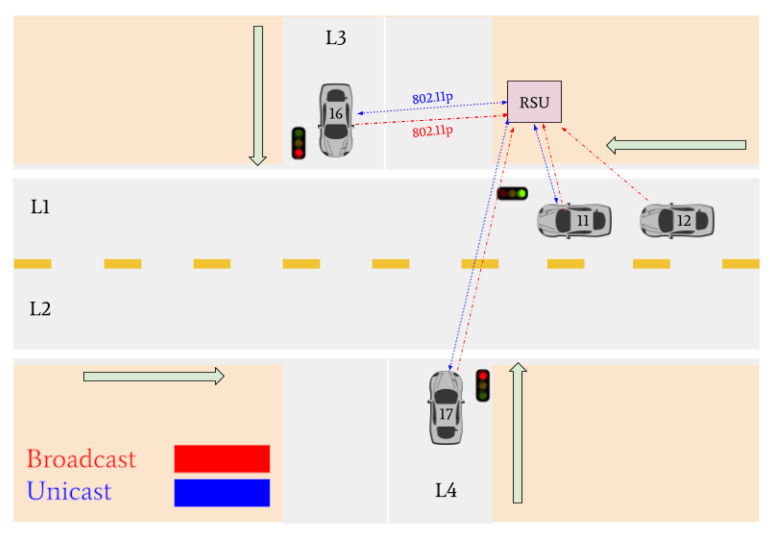
Vehicles 11, 16, and 17 arrive at the intersection and declare their intentions to the RSU. The RSU issues a green light to Vehicle 11 and red lights to vehicles 16 and 17. Vehicle 12 does not communicate its intentions since it is a non-lane leader.

**Figure 19:**
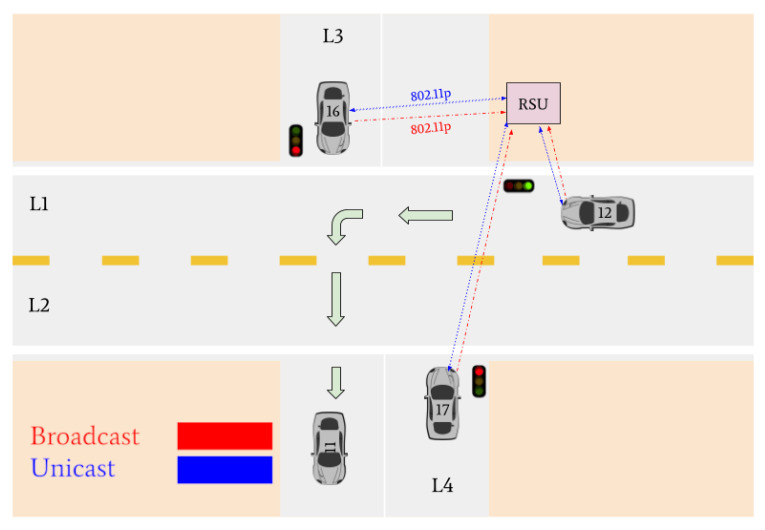
Vehicle 11 turns left. Vehicles 12, 16, and 17 declare their intentions to the RSU. The RSU issues a green light to Vehicle 12 and red lights to Vehicles 16 and 17.

**Figure 20:**
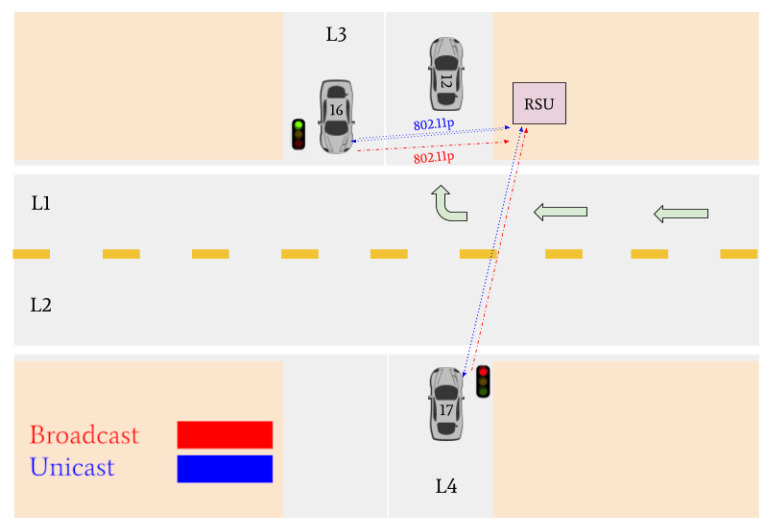
Vehicle 12 turns right. Vehicle 17 and Vehicle 16 declare their intention to the RSU. The RSU issues a green light to Vehicle 16 and a red light to Vehicle 17.

**Figure 21:**
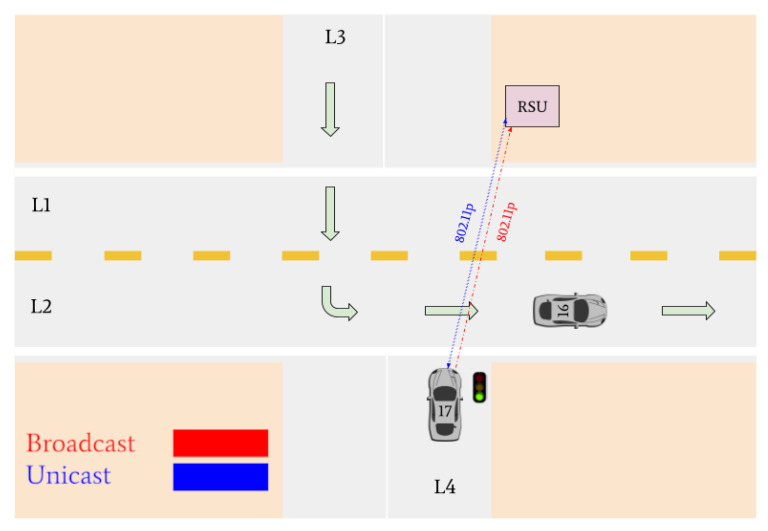
Vehicle 16 turns right. Vehicle 17 receives a green light from the RSU to turn right.

**Figure 22:**
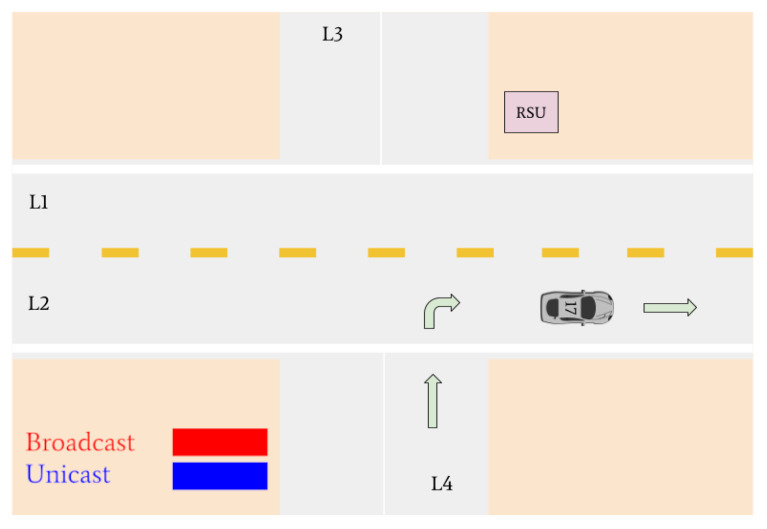
Vehicle 17 is the last remaining vehicle in the intersection and turns right.

**Figure 23:**
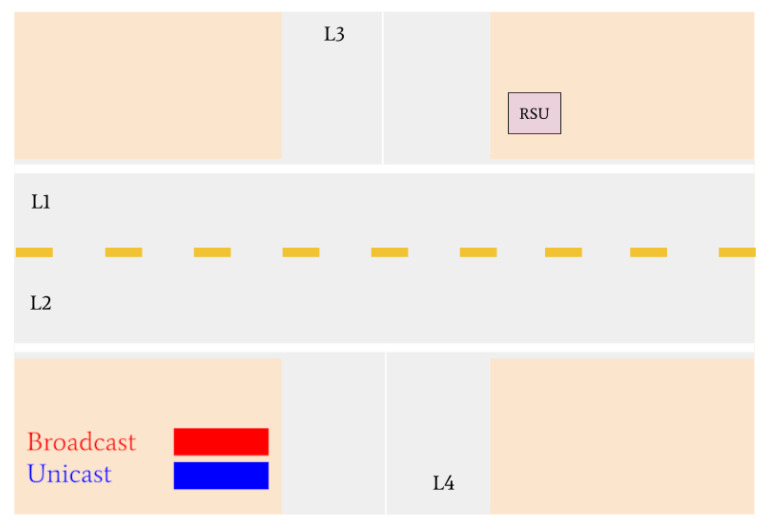
All vehicles have left the intersection and the simulation is complete.

Figure 17 indicates that Vehicle 17 is the only remaining vehicle at the intersection. Using unicast messages, the RSU determines that Vehicle 17 intends on turning right and issues it a green light. The intersection is then empty. A visual realization of the communications shown in Figure 15, Figure 16 and Figure 17 is presented in Figure 18, Figure 19, Figure 20, Figure 21, Figure 22 and Figure 23.

### 5.2. Network Metrics

**Figure 24:**
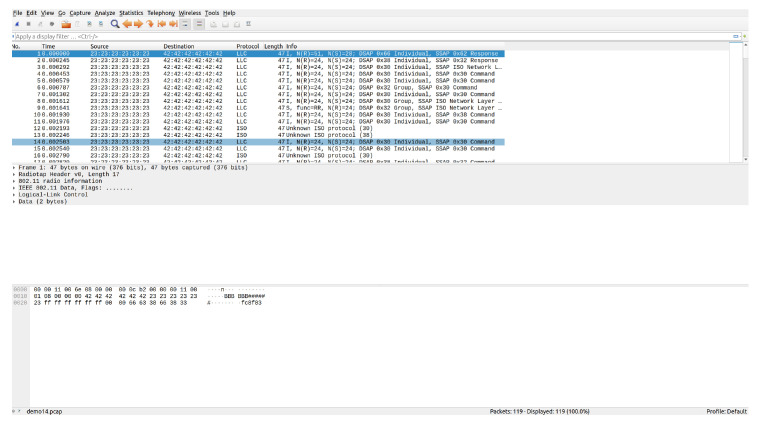
IEEE 802.11p messages received in Wireshark.

The packets received are saved to a pcap file, which can then be read by Wireshark. The messages sent are displayed with the header information followed by the data in the frame. Figure 24 displays a sample resulting file of running the program then transferring the messages through the HackRF Ones encapsulated in 802.11p. The entire message consists of 47 bytes (376 bits), with only 6 bytes of data.

**Figure 25:**
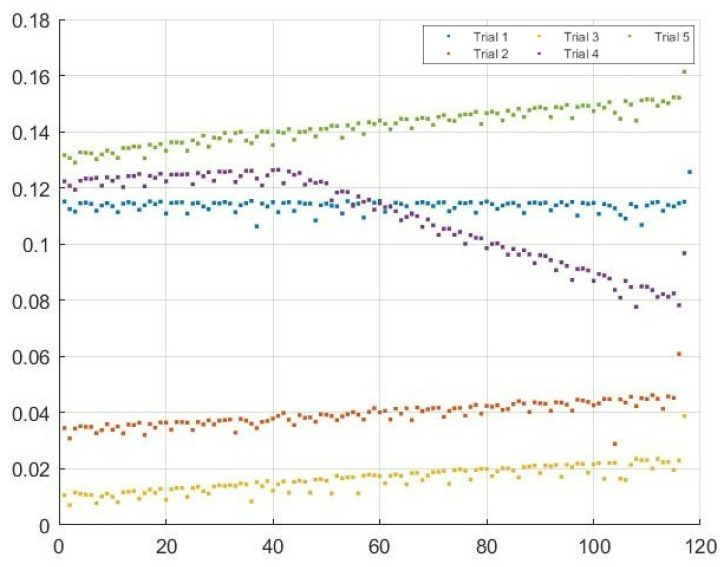
Delay in seconds of 120 packets transmitted during five trials over the Ethernet connection to the host GNU radio transmitter.

**Figure 26:**
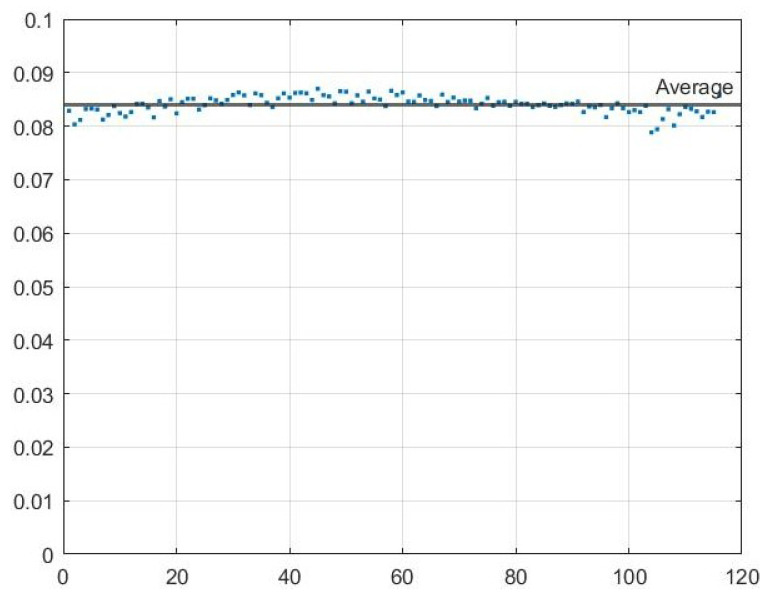
Flow-graph of average delay in seconds of 120 packets transmitted during five trials over the Ethernet connection to the host GNU radio transmitter.

**Figure 27:**
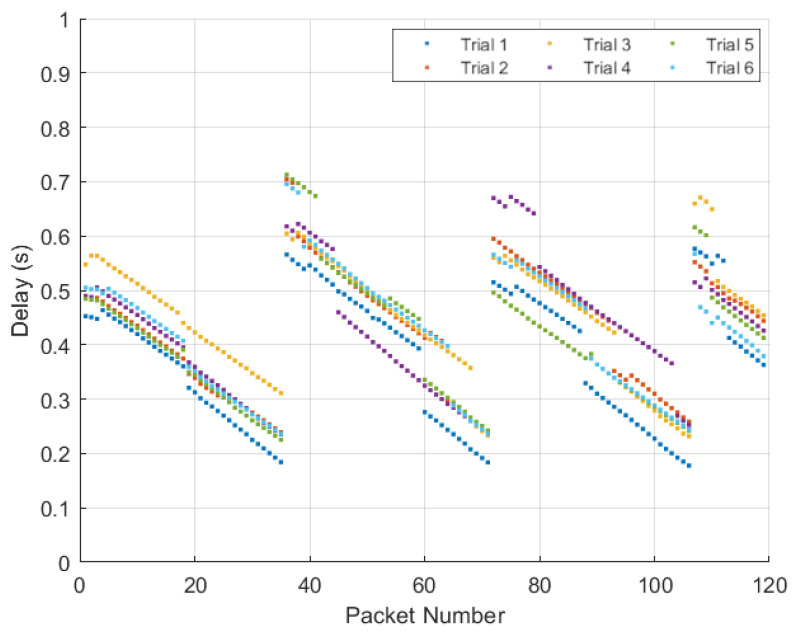
Delay in seconds over the IEEE 802.11p connection from the transmitting HackRF to the receiving HackRF for the 120 packets transmitted over six trials.

**Figure 28:**
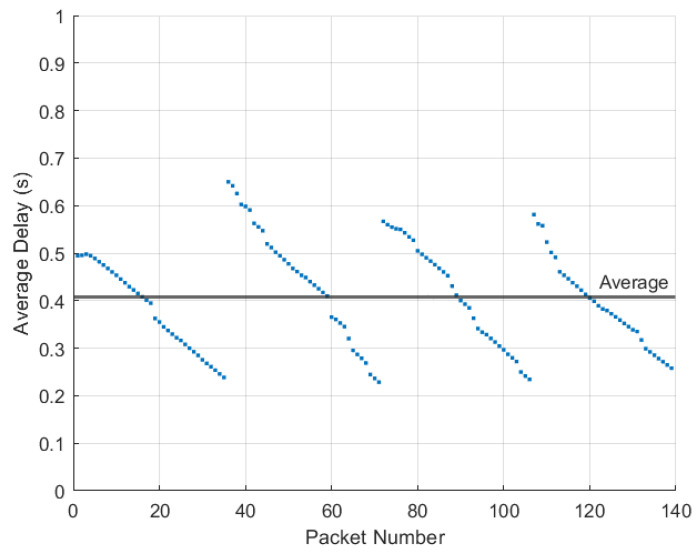
Flow-graph of average delay in seconds of the 120 packets transmitted during five trials over the 802.11p transmission from HackRF to HackRF.

An important metric in vehicular networks is delay, as vehicles are moving at high speeds. To examine the delay between the transmission and reception, 120 packets were sent five times and the delay was measured for each packet. The average delay for each packet was calculated as well as the the average delay across all packets. Figure 25 shows the results of this test for the delay between the Raspberry Pi ZMQ server and the ZMQ server on the computer hosting the GNU Radio transmission. Note the average delay across all packets of 0.0841 s. Figure 26 shows the results of this test for the delay between the transmitting HackRF and receiving HackRF. There is an interesting pattern in the delay since GNU Radio waits to receive multiple packets before transmitting which results in non-constant delay. The average delay added between the transmitting and receiving HackRF radios was 0.4067 s with a minimum of 0.2285 s (refer to Figure 27 and Figure 28).

Another metric considered important for vehicular communication is reliability. Across the entire end-to-end communication, 98.611% of the packets were received correctly, i.e., 118 packets on average for each trial. The majority of the trials had dropped a single packet, which was found to be the same packet of information each time. In addition to dropping one packet, in the first trial, four packets were received with errors. Since the same packet was dropped in each of the trials, it is likely due to a systematic error with the GNU Radio buffer. Further, the four erroneous packets occurred only in the first trial and not in the other five; this is likely caused by the setup of the initial trial rather than a flaw in the proposed system.

### 5.3. Power Data & Calculations

The battery size for the provided solar power system was 12 V and 5 Ah. The energy contained in this battery can be calculated as

(2) $$\begin{array}{cc} \left(\mathrm{Voltage})\times (\mathrm{Charge}\;\mathrm{Capacity}\right) & =(\mathrm{Energy}\;\mathrm{Capacity})\\ \left(12\;V)\times (5\;\mathrm{Ah}\right) & =60\;\mathrm{Wh}. \end{array}$$

The power consumption of each device in the system was measured using a multimeter to determine current, voltage, and power. These results are documented in Table 1. The total system power consumption based on these measurements was 11.85 W. Since the RSU will be operating continuously, the battery lifetime is given by

(3) $$\frac{60\;\mathrm{Wh}}{11.85\;W}=5.06\;h.$$

It is important to note that the recorded power data is for the prototype of the design, and it will change in an implementation using non-developmental equipment. The recommend system backup time for critical infrastructure is at least 10 days per the IEEE 1562 standard. A backup time of 5.06 h is not acceptable for an RSU since it is critical infrastructure, but this can be corrected by purchasing larger batteries and solar panels. The correct sizing of the panels and backup batteries can be determined from the IEEE 1013 and 1562 standards that specify sizing guidelines for stand-alone photovoltaic systems.

## 6. Conclusions

This paper proposed using an adapted virtual traffic light algorithm to reduce visibility issues of blind intersections in rural areas. The RSU-VIM algorithm is implemented on a prototype solar power-based RSU that communicates to vehicles following the IEEE 802.11p wireless communication standard. The proposed RSU-VIM over 802.11p system was validated through experimentation via comparatively inexpensive developmental equipment and hardware and the use of software defined radios and GNU radio with a message buffer. The results demonstrated its effectiveness with a simulated cloud implementation resulting in a delay of 0.0841 s and a total system delay of 0.4067 s and a 98.611% reliability.

The proposed system centralizes intervehicular communications into a single module that may be managed alongside other RSU modules. The ease of modularity enables flexible design and enhanced ability to manage the traffic system. Further, by offloading the computations onto the RSU while utilizing existing vehicular communications (i.e., broadcast messages), the module minimizes the additional use of limited resources and strain on individual vehicles.

Limitations of this system include: an increase in delay due to the nature of GNU radio software, the use of randomly generated traffic data, as well as that of simplified equipment meant for prototype creation. Future works include experimentation with real-world intersection data and using specialized equipment dedicated to optimizing the proposed RSU-VIM.

## Figures and Tables

**Table 1:** Current and voltage requirements for project components.

Component	Current (A)	Voltage (V)	Power (W)
Raspberry Pi	0.58	5.0	2.9
DE2-115 and Inverter	0.67	12.31	8.25
HackRF One	0.14	5.0	0.7
Total			11.85

## Data Availability

Not applicable.

## Abbreviations

The following abbreviations are used in this manuscript:

BSS	Basic Service Set
CAN	controller area network
CAV	Connected and Autonomous Vehicle
DSRC	Dedicated Short-Range Communication
FCC	Federal Communications Commission
FPGA	Field-Programmable Gate Array
GPIO	general purpose input/output
ICMP	Internet Control Message Protocol
IEEE	Institute of Electrical and Electronics Engineers
IoV	Internet of Vehicles
ITS	Intelligent Transportation Systems
LiDAR	light detection and ranging
OCB	Outside the Context of a BSS
radar	radio detection and ranging
RSU	Road-side Unit
RSU-VIM	Road-Side Unit-based Virtual Intersection Management
SAE	Society of Automotive Engineers
SDR	Software Defined Radio
sonar	sound navigation and ranging
V2I	vehicle-to-infrastructure
V2R	vehicle-to-roadside unit
V2V	vehicle-to-vehicle
V2X	vehicle-to-everything
WAVE	Wireless Access in Vehicular Environments
